# Acalabrutinib in Membranous Nephropathy Associated With Chronic Lymphocytic Leukemia

**DOI:** 10.1016/j.ekir.2022.09.004

**Published:** 2022-09-12

**Authors:** Hassan Izzedine, Isabelle Brocheriou, Alexis Mathian, David Ghez, Zahir Amoura

**Affiliations:** 1 Department of Nephrology, Peupliers Private Hospital, Paris, France; 2 AP-HP, Pitie Salpetriere Hospital, Department of Pathology, Paris, France; 3 AP-HP, Pitie Salpetriere Hospital, Department of Internal Medicine, Paris, France; 4 Gustave Roussy Institute, Department of Hematology, Villejuif, France

To the Editor:

Primary membranous nephropathy (pMN) is an autoimmune disease associated with autoantibodies targeting the phospholipase A2 receptor (PLA2R) in 80% of cases. Optimal treatment of pMN remains controversial. Rituximab, the first-line intravenous administered regimen in pMN according to Kidney Disease: Improving Global Outcomes^1^ is associated with high relapse rates.^2^

We report the case of a 58-year-old male patient with a history of stage I membranous nephropathy (MN) diagnosed in 2004. Partial remission was obtained with high-dose steroids and cyclophosphamide, with a 1 g per day residual proteinuria on irbesartan 150 mg per day. Fifteen years later in 2019, the appearance of a lymphocytosis (15 G/l) led to the diagnosis of Binet stage A chronic lymphocytic leukemia (CLL) requiring no immediate intervention. In September 2020, he presented with recurrence of nephrotic syndrome with edema, proteinuria 9 g per day, and hypoalbuminemia 27 g/l. Kidney biopsy revealed stage III IgG4 and PLA2R-positive MN (Figure 1a). Immunological testing revealed antinuclear antibody (Ab) at 1:320, anti-PLA2R Ab at 25U (N<10) without anti-THSD7A Ab.

**Figure 1:**
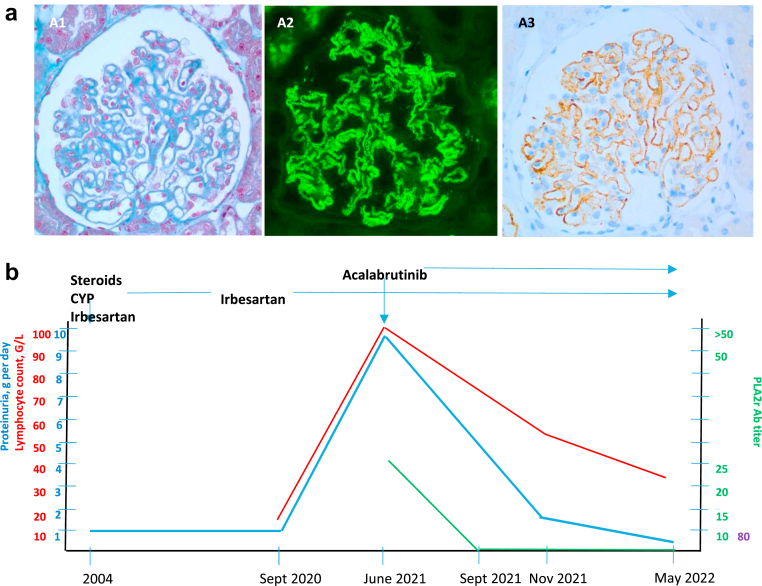
BTKi in Membranous Nephropathy. Kidney biopsy findings (a) Prominent thickening of the glomerular basement membrane with a hint of a vacuolated appearance. Little increase in mesangial hypercellularity (Masson’s trichrome; original magnification ×400, a1); strong and diffuse granular IgG staining along the glomerular capillaries without mesangial staining (Immunofluorescence, original magnification ×400, a2), mainly IgG4 (data not show), and intense granular staining of the capillary walls for PLA2R (Immunohistochemistry, original magnification x400, a3). Follow-up (b) Rapid clearance of anti-PLA2R Ab and marked decrease of proteinuria following acalabrutinib treatment, despite only partial control of lymphocyte count. BTKi, Burton’s tyrosine kinase inhibitor; CYP, cyclophosphamide.

Because the recurrence of MN coincided with CLL progression (white blood cell count 101 G/l, lymphocyte count 95 G/l), second-generation Burton’s tyrosine kinase inhibitor acalabrutinib 100 mg 2 times a day was initiated. Anti-PLA2R Ab became undetectable in 3 months, which was followed by the resolution of the nephrotic syndrome after 6 months. After 11 months on acalabrutinib, MN remains in remission (proteinuria < 0.5 g/d, serum albumin 3.45 g/dl) whereas CLL is in partial response with lymphocytosis (35 G/l) (Figure 1b).

The association between PLA2R Ab positive MN and malignancies have been described although the causal relationship in PLA2R Ab positive MN is debated.^2^^,^^3^ Patients with CLL present with immune disturbances, which constitute a notable feature of the disease compared to other chronic lymphoproliferative disorders, including frequently autoimmune cytopenia (e.g., autoimmune hemolytic anemia, thrombocytopenia, and pure red cell aplasia) and to a lesser extent nonhematological autoimmune diseases.^4^ One can speculate on the possibility that MN could be another autoimmune association. Indeed, rare cases of MN associated with CLL have been reported, some responding to B-cell-directed chemotherapy like fludarabine.^5^^,^S1

In our case, the rapid clearance of anti-PLA2R Ab following acalabrutinib treatment, despite only partial control of CLL, favors the hypothesis that acalabrutinib had a direct role in controlling the immune dysregulation, a frequent feature of CLL, leading to the production of pathogenic autoantibodies and might be an interesting alternative strategy in antibodies-mediated kidney diseases like pMN.

Randomized clinical trials are needed to assess the effectiveness of Burton’s tyrosine kinase inhibitors in the management of primary MN such as the ongoing enrollment phase study evaluating the efficacy and safety of SHR1459 in patients with pMN (Clinicaltrials.gov)

## Supplementary Material

Supplementary File (PDF)

**Supplementary Reference.**
