# Supplementary material for: Acalabrutinib in Membranous Nephropathy Associated With Chronic Lymphocytic Leukemia
Source: Kidney Int Rep. 2022 Sep 12;7(11):2539–40. doi: 10.1016/j.ekir.2022.09.004 (PMC9751673; doi:10.1016/j.ekir.2022.09.004)
Supplement: Supplementary File (PDF) [file mmc1.pdf]

Supplementary Reference

S1. Strati P, Nasr SH, Leung N, et al. Renal complications in chronic lymphocytic leukemia and monoclonal B-cell lymphocytosis: the Mayo Clinic experience. *Haematologica*. 2015;100:1180-1188. doi:10.3324/haematol.2015.128793
